# Evaluation of Two Triplex One-Step qRT-PCR Assays for the Quantification of Human Enteric Viruses in Environmental Samples

**DOI:** 10.1007/s12560-017-9293-5

**Published:** 2017-04-08

**Authors:** Kata Farkas, Dafydd E. Peters, James E. McDonald, Alexis de Rougemont, Shelagh K. Malham, Davey L. Jones

**Affiliations:** 1 School of Environment, Natural Resources and Geography, Bangor University, Deiniol Road, Bangor, Gwynedd LL57 2UW UK; 2 School of Medical Sciences, Bangor University, Brigantia Building, Penrallt Road, Bangor, Gwynedd LL57 2AS UK; 3 School of Biological Sciences, Bangor University, Deiniol Road, Bangor, Gwynedd LL57 2UW UK; 4 Centre National de Référence Des Virus Entériques, Laboratoire de Virologie-Sérologie, CHU de Dijon, 2 rue Angélique Ducoudray, BP 37013, 21070 Dijon Cedex, France; 5 UMR PAM A 02.102 Procédés Alimentaires et Microbiologiques, Université de Bourgogne Franche-Comté/AgroSup Dijon, 1 Esplanade Erasme, 21000 Dijon, France; 6 School of Ocean Sciences, Bangor University, Menai Bridge, Anglesey LL59 5AB UK

**Keywords:** Multiplex real-time reverse transcription PCR, Nucleic acid quantification, Enteric viruses, Sediment, Shellfish

## Abstract

Human enteric viruses are responsible for waterborne and shellfish-associated disease outbreaks worldwide. Quantitative reverse transcription PCR (qRT-PCR) is often used to assess the health risks associated with shellfish and environmental water, but viral titres in sediments are less commonly investigated. In this study, we developed and validated two multiplex qRT-PCR assays for aquatic sediment and shellfish samples targeting viruses that are a common cause of gastroenteritis (norovirus GI, GII and hepatitis A virus), two emerging viruses (sapovirus and hepatitis E virus), along with mengovirus (MgV), which is often used as a sample process control for the assessment of RNA extraction efficiency. Singleplex and multiplex assays demonstrated comparable PCR efficiencies and gave reliable results over a wide concentration range. The multiplex assays showed remarkable sensitivity with a limit of detection of 1 RNA copy/µL nucleic acid extract for all target viruses and limits of quantification of 3–18 RNA copies/µL for the targeted human pathogenic viruses and 20–40 RNA copies/µL for MgV. The results demonstrated the veracity of multiplex qRT-PCR for the estimation of viral titres in sediment and shellfish, allowing the rapid assessment of viral infection risks associated with environments exposed to wastewater contamination.

## Introduction

Enteric viruses represent a major risk to human health, being responsible for numerous local and global disease outbreaks. For example, noroviruses (NoV) are responsible for approx. 3 million acute gastroenteritis cases in the UK and 20 million cases in the USA each year (Centers for Disease Control and Prevention 2015; Tam et al. 2012) placing an enormous burden on health care systems and the wider economy. Hepatitis A and E viruses (HAV and HEV), causing acute liver disease, have also been shown to be associated with large disease outbreaks. HAV used to be the most frequent cause of hepatitis worldwide; however, with improved sanitation and vaccination it has become less frequent in developed countries (Tahaei et al. 2012). Nonetheless, HAV, along with NoV, still represents the major cause of foodborne viral outbreaks (Bosch et al. 2016), including those associated with shellfish (Bellou et al. 2013). HEV was responsible for the largest viral waterborne outbreak in New Delhi in December 1955–January 1956, where more than 29,000 cases were registered with HEV infection (Purcell 1996; Rao and Melnick 1986). Recently, HEV and sapoviruses (SaV), which cause gastroenteritis similar to NoV infection, have been responsible for sporadic cases in developed countries and are now considered as emerging pathogens (Dalton et al. 2013; Yates 2014).

Due to the high risks associated with the consumption of shellfish contaminated with enteric viruses, a standard method is now available for the quantification of NoV GI, GII and HAV (International Organization for Standardization 2013; Lees and CEN WG6 TAG4 2010). This method recommends the use of a one-step quantitative reverse transcription PCR (qRT-PCR) for the enumeration of viral RNA in the extract of shellfish digestive gland. However, while this approach does not give information on the infectivity status of the virus particles, it is the most sensitive and accurate method available for those viruses whose propagation in vitro is challenging, e.g. human NoV and SaV. Recent efforts have also been made to use molecular methods for enteric virus detection in sediment (Miura et al. 2011; Staggemeier et al. 2015a, b). Nonetheless, these methods often lack thorough validation, and the recoveries may vary due to the contrasting chemical composition of the samples. Therefore, the use of extraction and sample process controls (such as mengovirus) is recommended (Hennechart-Collette et al. 2015; International Organization for Standardization 2013; Martin-Latil et al. 2012); however, they are not routinely used.

The lack of proper validation and use of controls may be associated with the high costs and extended labour of the individual quantification of RNA viruses using qRT-PCR. Methods are available for the quantification of enteric viruses from shellfish, water and faecal samples using duplex and multiplex assays (Farkas et al. 2015; Fuentes et al. 2014; Lee et al. 2016; Martin-Latil et al. 2012; van Maarseveen et al. 2010; Yan et al. 2013); however, no method has been validated for sediment. In this study, we validated two triplex qRT-PCR assays for the simultaneous quantification of the RNA of the most prevalent NoV genogroups (GI and GII), HAV, two emerging pathogens, SaV GI and HEV, and a commonly used extraction control, mengovirus (MgV), in sediment and shellfish nucleic acid extracts. One assay targeted the NoV GI, SaV and HEV and the other assay targeted the NoV GII, HAV and MgV.

## Materials and Methods

### Target Viral RNA

NoV GI RNA, MgV strain VMC0 and HAV strain pHM17543c were kindly provided by Dr. Lisa Cross (Centre for Environment Fisheries and Aquaculture Science; CEFAS UK). NoV GI RNA was derived from a pool of nucleic acid extracts of shellfish samples processed according to the ISO/TS 152016-1 (2013) standard. The sample was tested for all viruses targeted in this study using the singleplex qRT-PCR assays detailed below and contained no other target viruses. SaV GI.2 was obtained from clinical stool samples and genotyped by the National Reference Centre for Enteric Viruses, Dijon, France. The viral sample was generated by the preparation of a 10% solution, using phosphate-buffered saline (pH 7.4), which was subsequently filtered through a 0.2-µm filter. Norovirus sample (diluted and filtered faecal matter from a patient with confirmed NoV GII infection) and HEV RNA (ORF3 segment) were provided by Prof. Ian Goodfellow (University of Cambridge, UK). When necessary, viral RNA was extracted from a 0.5 mL viral sample using the NucliSENS^®^ MiniMag^®^ Nucleic Acid Purification System (bioMérieux SA, France) and eluted in 100 µL molecular grade water. Viruses and viral RNA were stored at −80 °C. Nucleic acid extracts derived from faecal matter were tested for all viruses targeted in this study using the singleplex qRT-PCR assays detailed below. Results confirmed that the samples contained no target virus other than NoV GII or SaV GI.

### Spiking Environmental Samples

Sediment and mussel samples were collected in the Conwy estuary (53°17′37.5″N 3°50′22.0″W), North Wales, at low tide. A sediment sample was processed using the elution–concentration method described elsewhere (Farkas et al. 2017; Lewis and Metcalf 1988). In brief, five aliquots of 10 g sediment sample were mixed with 30 mL 3% beef extract in 2 M NaNO_3_ (pH 5.5) for 30 min and the solid matter was removed by centrifugation at 2500 × *g* for 10 min. The pH of the eluent was adjusted to 7.5, then incubated in 15% polyethylene glycol 6000 and 2% NaCl overnight at 4 °C and centrifuged at 10,000 × *g* for 30 min at 4 °C. The digestive tissue (DT) of 30 mussels was extracted and homogenised. Aliquots of 2 g of the digestive tissue mix were treated with proteinase K according to the ISO/TS 152016-1 (2013) standard. Viral nucleic acids of the sediment and shellfish DT concentrates were extracted using the NucliSENS^®^ MiniMag^®^ Nucleic Acid Purification System (bioMérieux SA, France). Preliminary findings confirmed that the samples were negative for all target viruses prior to spiking. A pool of viral nucleic acids (NoV GI and GII, SaV, HAV, HEV, MgV) were added to the nucleic acid extracts at the ratio of 1:50 to reach a final concentration of approx. 10^6^ RNA copies/µL. That sample was further diluted in sediment or shellfish extract to achieve the final concentrations of 10^5^, 10^4^, 10^3^, 10^2^, 60, 40, 20, 10, 5 and 1 RNA copies/µL.

### qRT-PCR Assay

All qRT-PCR assays were carried out in a QuantStudio™ Flex 6 Real-Time PCR System (Applied Biosystems, USA). Standards for NoV GI, GII and HAV (prepared according to ISO/TS 15216-1:2013 standard) were kindly provided by Dr. Lisa Cross (CEFAS, UK). SaV and MgV standards were derived from cloning qRT-PCR amplicons into pGem-T Easy vector (Promega, USA). A pSV plasmid incorporating the HEV ORF3 gene was kindly provided by Prof. Ian Goodfellow (University of Cambridge, UK). Plasmids were transformed to Alpha Select Bronze Competent Cells (Bioline, UK) and isolated using the ISOLATE II Plasmid Mini Kit (Bioline, UK). Recombinant plasmids were quantified using NanoDrop ND-1000 (NanoDrop, USA) and 10-fold serially diluted. Standard dilutions ranging from 10^5^ to 10^0^ DNA copies/µL in triplicate were used to generate standard curves for qRT-PCR quantification. Amplification efficiency, slope and R^2^ were determined based on the standard curve of each reaction and calculated by the QuantStudio™ Real-time PCR software (Applied Biosystems, USA).

Primers and probes are listed in Table 1. All primers and probes were adapted from previous studies; however, the reporters and/or quenchers were replaced for multiplex applications.

**Table 1:** Primers and probes used for the singleplex/multiplex qRT-PCR assays

Virus	Primers/probes	Sequence (5′–3′)	Amplicon length	References
Assay 1				
Norovirus GI	QNIF4-F	CGCTGGATGCGNTTCCAT	86 bases	Da Silva et al. (2007)
	NV1LC-R	CCTTAGACGCCATCATCATTTAC		Svraka et al. (2007)
	TM9-P	FAM-TGGACAGGAGATCGC-NFQMGB		Hoehne and Schreier (2006)
Sapovirus GI	CU-SV-F	TTGGCCCTCGCCACCTAC	101 bases	Chan et al. (2006)
	CU-SV-R	CCCTCCATYTCAAACACTAWTTTG CAAATTAGTGTTTGAGATGGAGGG		Chan et al. (2006)
	CU-SV-P	VIC-TGGTTCATAGGTGGTAC-NFQMGB*		Chan et al. (2006)
Hepatitis E virus	JVHEV-F	GGTGGTTTCTGGGGTGAC	71 bases	Jothikumar et al. (2006)
	JVHEV-R	AGGGGTTGGTTGGATGAA		Jothikumar et al. (2006)
	JVHEV-P	ABY-TGATTCTCAGCCCTTCGC-QSY*		Jothikumar et al. (2006)
Assay 2				
Norovirus GII	QNIF2-F	ATGTTCAGRTGGATGAGRTTCTCWGA	89 bases	Loisy et al. (2005)
	COG2-R	TCGACGCCATCTTCATTCACA		Kageyama et al. (2003)
	QNIFS-P	FAM-AGCACGTGGGAGGGCGATCG-QSY		Loisy et al. (2005)
Hepatitis A virus	HAV68-F	TCACCGCCGTTTGCCTAG	173 bases	Costafreda et al. (2006)
	HAV240-R	GGAGAGCCCTGGAAGAAAG		Costafreda et al. (2006)
	HAV150-P	VIC-CCTGGACCTGCAGGAATTAA-QSY*		Costafreda et al. (2006)
Mengovirus	Me110-F	GCGGGTCCTGCCGAAAGT	100 bases	Pinto et al. (2009)
	Me209-R	GAAGTAACATATAGACAGACGCACAC		Pinto et al. (2009)
	Me147-P	ABY-ATCACATTACTGGCCGAAGC-NFQMGB*		Pinto et al. (2009)

*F* forward primer, *R* reverse primer, *P* probe, *FAM* 6-Carboxyfluorescein, *NFQMGB* non-fluorescent quencher/minor groove binder

*Original fluorescent dyes used for probes were replaced for multiplex assay development

All singleplex and multiplex qRT-PCR assays were based on a single-step TaqMan-based assay described in the ISO/TS 15216-1:2013 standard (International Organization for Standardization 2013) using the RNA UltraSense One-step qRT-PCR kit (Invitrogen, USA). The 20 μL qRT-PCR reaction mix contained 1xRNA UltraSense Reaction Mix with 1 µL RNA  UltraSense Enzyme Mix, 10 pmol of the forward and the reverse primers, 5 pmol of the probe/probes, 0.1 × ROX reference dye,  1 µg bovine serum albumin (BSA) and 3 μL of the sample/standard. Negative controls (3 µL molecular grade water) were included in each run. Due to the differences in the melting temperatures of the primers and probes, two assays were validated: one with the annealing temperature (Ta) of 56 °C for the detection of NoV GI, SaV and HEV and another with Ta of 60 °C for NoV GII, HAV and MgV. The qRT-PCR assay consisted of a 60-min reverse transcription step at 55 °C followed by a 5-min step of denaturation at 95 °C, and 45 cycles of amplification consisting of 95 °C for 15 s, 56 °C or 60 °C for 60 s and 65 °C for 60 s. The baseline (cycle threshold; Ct) was manually adjusted after each run.

### Reproducibility and Sensitivity of the Multiplex qRT-PCR Assay

To investigate reproducibility, the serial dilutions of spiked sediment and shellfish extracts were assayed using qRT-PCR on two plates in duplicate on each plate. The nominal concentrations of the spiked samples were 10^5^, 10^4^, 10^3^, 10^2^ and 10^1^ RNA copies/µL. To avoid RNA degradation, dilution series were freshly prepared before each run.

The limit of detection (LOD) and the limit of quantification (LOQ) were estimated according to the EN 24790 guideline (European Network of GMO laboratories 2011). Replicates of ten of the shellfish and sediment samples spiked with viral RNA were used at the nominal concentrations of 60, 40, 20, 10, 5 and 1 RNA copies/µL in each singleplex and multiplex assay. In order to determine RNA concentration in the samples, dilution series of the plasmid standards covering the concentration range of the samples were used. The lowest concentration where all replicates were positive was the estimated LOD. LOQ was estimated as the lowest concentration where the coefficient of variation (CV) amongst replicates was below 0.25.

## Results

### Multiplex Assay Performance

**Fig. 1:**
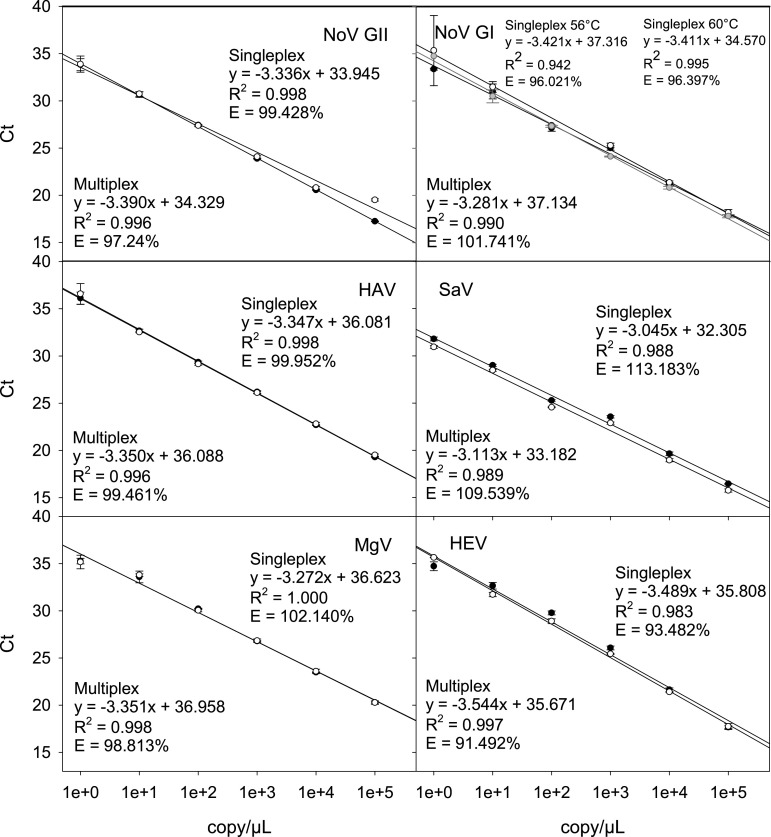
Standard curves of the qRT-PCR assays for the target viruses in singleplex (*black* *filled circle*) and multiplex (*open circle*) qRT-PCR assays. Norovirus GII (NoV GII), hepatitis A virus (HAV and mengovirus (MgV) assays were run using the annealing temperature (Ta) of 60 °C, whereas NoV GI, sapovirus (SaV) and Hepatitis E virus (HEV) assays were run using Ta of 56 °C. The *grey circle* represents the results for the norovirus GI standards when a Ta of 60 °C was used. *Symbols* and *error bars* represent the mean and standard deviation of the triplicated experiments

For each virus type, dilution series of plasmid standards incorporating the target genes were used for validation and quantification in singleplex and multiplex qRT-PCR assays. All standards showed excellent negative linearity in the range tested in both singleplex and multiplex assays (Fig. 1). The *R* ^2^ values ranged from 0.942 to 1 and the assay efficiency (E) ranged from 91.5 to 113.2%. The difference in these values were negligible between multiplex and singleplex assays. The corresponding standard curves overlapped in most cases, except in the SaV assays where lower Ct values were observed for all dilutions in the multiplex assay than in the singleplex.

### Validation with Spiked Sediment and Shellfish Extract Samples

The usefulness of the singleplex and multiplex qRT-PCR assays for environmental samples was assessed using a serial dilution of sediment and shellfish nucleic acid extracts spiked with known concentration of the RNA of target viruses. Samples were run in duplicate in two assays to assess intra- and inter-assay variability. All samples tested were positive except the lowest concentration of MgV sample in shellfish extract, which was negative in both singleplex assays, but positive in the multiplex runs (Table 2). The CV values, based on the RNA copy concentration and standard deviation of duplicate samples in two assays (*n* = 4), were all lower than 0.5 in the high-concentration samples, except those calculated for the NoV GII in the sediment extracts and for HAV with the concentration of approx. 1000 copies/µL. The high variation noted for those samples may have been a result of pipetting error during sample preparation. Higher CV values were noted for SaV and HEV with <400 copies/µL and for other viruses with concentration <100 copies/µL.

**Table 2:** Comparison of singleplex and multiplex qRT-PCR assays using sediment and shellfish extracts spiked with known concentration of viral RNA

Virus	Dilution factor	Sediment extract				Shellfish extract			
		Singleplex		Multiplex		Singleplex		Multiplex	
		Concentration mean ± SD	CV	Concentration mean ± SD	CV	Concentration mean ± SD	CV	Concentration mean ± SD	CV
		RNA copies/µL		RNA copies/µL		RNA copies/µL		RNA copies/µL	
NoV GII	1	35,393 ± 20,300	0.574	41,213 ± 26,660	0.647	57,960 ± 20,548	0.355	43,000 ± 3809	0.089
	2	3490 ± 1536	0.440	4632 ± 2720	0.587	6730 ± 1636	0.243	5623 ± 368	0.065
	3	424 ± 214	0.505	522 ± 273	0.522	648 ± 122	0.188	714 ± 127	0.178
	4	55 ± 46	0.838	85 ± 74	0.866	66 ± 6	0.091	72 ± 23	0.326
	5	10 ± 4	0.413	7 ± 4	0.593	7 ± 2	0.267	8 ± 6	0.662
HAV	1	42,371 ± 5614	0.133	43,312 ± 8557	0.198	58,282 ± 1416	0.024	47,415 ± 5283	0.111
	2	5890 ± 205	0.035	5736 ± 899	0.157	6278 ± 343	0.055	4672 ± 605	0.129
	3	1164 ± 849	0.729	1178 ± 704	0.597	1259 ± 881	0.700	1400 ± 855	0.610
	4	111 ± 85	0.771	96 ± 45	0.468	117 ± 84	0.721	99 ± 81	0.816
	5	10 ± 5	0.555	8 ± 5	0.657	11 ± 7	0.616	8 ± 4	0.475
MgV	1	69,443 ± 3835	0.055	77,814 ± 11,940	0.153	63,339 ± 426	0.007	89,943 ± 55,537	0.617
	2	6814 ± 1119	0.164	8919 ± 396	0.044	8671 ± 2789	0.322	12,388 ± 666	0.054
	3	713 ± 127	0.178	949 ± 193	0.203	599 ± 77	0.128	632 ± 218	0.345
	4	82 ± 28	0.345	113 ± 10	0.091	110 ± 37	0.338	47 ± 11	0.223
	5	7 ± 6	0.807	16 ± 3	0.188	Not determined	Not determined	Not determined	
NoV GI	1	94,489 ± 2873	0.030	101,618 ± 14,852	0.146	92,346 ± 9730	0.105	84,602 ± 152	0.002
	2	10,148 ± 491	0.048	11,802 ± 3575	0.303	10,832 ± 156	0.014	11,853 ± 2560	0.216
	3	1131 ± 29	0.026	1271 ± 462	0.363	1178 ± 176	0.150	2898 ± 1184	0.409
	4	129 ± 5	0.038	161 ± 50	0.314	137 ± 11	0.080	153 ± 52	0.336
	5	29 ± 0	0.015	66 ± 52	0.786	17 ± 5	0.267	15 ± 11	0.703
SaV	1	44,359 ± 1813	0.041	34,540 ± 12,679	0.367	39,071 ± 13,819	0.354	40,095 ± 4704	0.117
	2	3773 ± 560	0.148	2994 ± 1353	0.452	3462 ± 1664	0.481	3742 ± 835	0.223
	3	313 ± 67	0.213	249 ± 139	0.560	288 ± 134	0.466	284 ± 76	0.266
	4	26 ± 8	0.314	21 ± 12	0.574	25 ± 15	0.583	25 ± 10	0.388
	5	2 ± 1	0.408	4 ± 4	1.031	2 ± 1	0.526	8 ± 8	1.066
HEV	1	25,270 ± 8022	0.317	21,512 ± 12,217	0.568	21,939 ± 3551	0.162	12,742 ± 620	0.049
	2	2738 ± 1362	0.498	2029 ± 1570	0.774	1923 ± 363	0.189	891 ± 129	0.145
	3	212 ± 108	0.511	120 ± 76	0.632	161 ± 32	0.199	81 ± 11	0.135
	4	19 ± 9	0.465	11 ± 6	0.513	18 ± 0	0.020	6 ± 1	0.228
	5	2 ± 1	0.375	1 ± 1	0.403	2 ± 0	0.058	1 ± 0	0.399

The LOD and LOQ for the multiplex assays were determined by running ten replicates of low concentrations of spiked sediment and shellfish extracts. The LOD for all virus types was 1 RNA copy/µL. The highest LOQs were observed in sediment and shellfish extracts spiked with MgV (20 and 40 copies/µL, respectively). The LOQ of the other target viruses ranged from 5 to 10 copies/µL in spiked sediment and from 3 to 18 copies/µL in shellfish samples (Table 3).

**Table 3:** Limit of quantification (LOQ) of target viral sequences in sediment and shellfish nucleic acid extracts

	Sediment		Shellfish	
	LOQ	CV	LOQ	CV
	RNA copies/µL extract	%	RNA copies/µL extract	%
NoV GI	9	0.119	18	0.195
SaV	9	0.249	9	0.232
HEV	8	0.210	12	0.240
NoV GII	5	0.207	13	0.200
HAV	10	0.194	3	0.194
MgV	20	0.178	40	0.153

LOQ was the lowest concentration where the CV among replicates was ≤0.25 (*n* = 10)

## Discussion

The accurate detection and quantification of a wide range of waterborne enteric viruses is essential to investigate health risks associated with wastewater contamination of environmental matrices. In this study, we evaluated the usefulness of two triplex one-step qRT-PCR assays targeting five strains of human enteric viruses (NoV GI/GII, HAV, HEV, SaV GI) and a murine cardiovirus (MgV), which is often used as an extraction control for shellfish sample processing.

All the primers and probes used have been described elsewhere (see Table 1 for references) and were shown to be specific to the target sequences. For some of the probes, the replacement of the published reporter and quencher dyes was necessary for multiplexing. For the SaV and HAV probes, the 6-carboxyfluorescein (FAM) reporter dye (fluorescent emission at 517 nm) was replaced with VIC (emission at 551 nm), and for the HEV and MgV probes it was replaced with ABY (emission at 580 nm). The TAMRA and the black hole quencher of the NoV GII and HEV probes were replaced with a non-fluorescent quencher, QSY. The replacements did not affect the melting temperature of the probes.

The Ta for the SaV and HEV assays was 56 °C (Chan et al. 2006; Jothikumar et al. 2006) and the primers and probes for those assays failed to align when the Ta was increased to 60 °C. Therefore, the Ta for the NoV GI assay was lowered to 56 °C from the reported value of 60 °C (International Organization for Standardization 2013) for multiplexing. Preliminary results based on the standard curves run at 56 and 60 °C showed no significant difference in PCR efficiency when the lower Ta was used (Fig. 1).

Performances of the singleplex and multiplex assays were first compared using a dilution series of DNA plasmids incorporating the target sequences. The overlapping standard curves suggested no competition for resources when multiplex approach was used. The only exception was the SaV assay where the standard curve of the multiplex assay slightly shifted (Fig. 1). However, the difference between the corresponding mean Ct values was below 1 and did not affect accurate quantification. Overall, the results suggested that all singleplex and multiplex assays are suitable for the quantification of the target sequences in a wide concentration range.

In order to further assess assay sensitivity and reproducibility, nucleic acid extracts of sediment and shellfish samples were spiked with known concentrations of viral RNA. The results suggested that the singleplex and multiplex assays were reproducible between approx. 10 and 10^5^ RNA copies/µL sample concentration, which refers to 50–5 × 10^5^ RNA copies/g sediment and 2 × 10^3^–2 × 10^7^ RNA copies/g shellfish assuming that the elution and nucleic acid extraction methods were used with 100% recoveries. These concentrations cover the range of viral nucleic acid concentration common in wastewater-contaminated sediment (Miura et al. 2011) and shellfish (Lowther et al. 2012). Assay sensitivity may be increased by increasing the sample volume or the volume of the qRT-PCR reaction mix (Le Mennec et al. 2016). Results revealed no inhibition due to residual organic matter in the nucleic acid extracts that has been shown to affect reverse transcriptase and polymerase enzymes (Farkas et al. 2017; Iker et al. 2013; Meschke and Sobsey 1998; Rock et al. 2010). The multiplex assay showed high sensitivity with a LOD of 1 RNA copy/µL. This refers to 10 RNA copies/g sediment and 200 RNA copies/g shellfish DT, assuming 100% recovery during viral elution and nucleic acid extraction. The LOQ values, varying between 3 and 40 copies/µL, allowed the accurate quantification of the samples tested.

The LOQ of multiplex qRT-PCR for the MgV was slightly higher (20 and 40 RNA copies/µL in sediment and shellfish extracts, respectively) than that observed for the enteric viruses. The MgV is usually added to a sample before extraction of the nucleic acids in known concentrations. According to the ISO/TS 152016-1 (2013), the extraction is considered successful when the recovery of the extraction control is above 1%, whereas the evaluation of the method used for the elution and concentration of viruses in sediment has shown recoveries above 70% (Farkas et al. 2017; Lewis and Metcalf 1988). Hence, we recommend the addition of 10^6^ intact MgV particles to samples prior to extraction when using the multiplex qRT-PCR (resulting Ct of 23-24) to assess process performance.

## Conclusions

The two multiplex qRT-PCR assays validated in this study allow the accurate quantification of the target viruses in sediment and shellfish nucleic acid extracts. Multiplexing enables the identification of different target pathogens in one assay, lowering the cost and time associated with qRT-PCR. The use of MgV as an extraction control allows the quality assessment of the extraction method used prior to qRT-PCR, lowering the possibility of false-negative outcomes. Overall, these assays allow the thorough assessment of the target viruses in sediment and shellfish samples and are useful for quantitative risk assessment of wastewater-contaminated environments.
